# Low serum levels of vitamin D are associated with anxiety in children and adolescents with dialysis

**DOI:** 10.1038/s41598-018-24451-7

**Published:** 2018-04-13

**Authors:** Bin Han, Fu-Xiang Zhu, Hai-Feng Yu, Si Liu, Jun-Liang Zhou

**Affiliations:** Department of nephrology, First Affiliated Hospital of Jiaxing University, Jiaxing, Zhejiang China

## Abstract

Anxiety is a frequent and serious complication of children and adolescents receiving dialysis. Low serum vitamin D levels have been associated with anxiety in non-pediatric patients. This study sought to examine the possible association between serum vitamin D levels and the presence of anxiety in children and adolescents with dialysis in China. A total of 156 pediatric patients who were on hemodialysis or peritoneal dialysis and 100 healthy controls were included in the current study. Serum 25-hydroxyvitamin D [25(OH)D] levels were measured by using a competitive protein-binding assay. Anxiety was assessed by using the Chinese version of the Screen for Child Anxiety Related Emotional Disorders (SCARED, = 25 as cutoff). Among 156 patients, 110 had a current anxiety (70.5%) and 46 did not (29.5%). Serum levels of 25(OH)D were significantly lower in patients with anxiety than in normal controls (19.4 ± 10.3 vs. 38.6 ± 15.5 ng/ml, *P* < 0.001). Serum 25(OH)D levels (≤15.0 ng/ml) were independently associated with the existent of anxiety in children and adolescents receiving dialysis (OR 4.650, 95% CI 1.663–13.001, *P* = 0.003). Our research demonstrates that low serum levels of vitamin D are independently associated with anxiety among children and adolescents on dialysis, which needs to be confirmed in future experimental and clinical studies.

## Introduction

Psychiatric disorders such as anxiety and depression are prevalent in children and adolescents with chronic kidney disease, especially in those on dialysis^1^. Anxiety, a part of emotional status, is one of the most frequent and important psychiatric complications among children and adolescents with dialysis^2,3^. The recognition and diagnosis of anxiety is of importance because the presence of anxiety has been associated with reduced quality of life and poor social relationships in pediatric patients receiving dialysis^4^. Compared with depression, however, data on prevalence and correlates of anxiety in children and adolescents with dialysis is limited.

Vitamin D, a neurosteroid hormone, is extremely essential for human health^5^. The profusion of vitamin D receptors (VDR) and vitamin D activating enzyme 1ahydroxylase in brain tissue suggest a key role of vitamin D in certain mental processes^6,7^. A growing body of evidence demonstrates an association between low vitamin D levels and anxiety in clinical populations^8–12^. Studies in animals have identified that mice lacking the vitamin D receptors gene showed an increase in anxiety-like behavior^13^. A recent study has showed a therapeutic effect of vitamin D on anxiety-like behavior in female rats with long-term estrogen deficiency^14^. Vitamin D deficiency/insufficiency is common in children and adolescents with dialysis^15,16^. To date, however, no study has examined the association between vitamin D and anxiety in children and adolescents with dialysis.

Therefore, the aim of the current study was to investigate the prevalence and correlates of anxiety in children and adolescents with dialysis in China. Moreover, we examined whether low levels of vitamin D are associated with anxiety in children and adolescents with dialysis.

## Results

### Baseline Characteristics of Study Samples

Of the 169 dialysis patients screened for study entry, we excluded individuals with missing information on serum levels of vitamin D (n = 8), with a history of depression and anxiety (n = 3), leukaemia (n = 1), and losing a family member in the past 30 days (n = 1). The final analysis included 156 patients (age 13.8 ± 2.4 years; 58% boys and 42% girls). Antihypertensive therapy was administered in 82.1% of dialysis patients. There were no significant differences in age and gender between the included patients and the excluded patients.

### Characteristics of the sample per tertile of serum 25-hydroxyvitamin D

Patients in the lowest tertile (≤15.0 ng/ml) were more likely to be girl (*P* = 0.003) and had higher intact parathyroid hormone (iPTH) (*P* < 0.001) and higher Screen for Child Anxiety Related Emotional Disorders (SCARED) scores (*P* = 0.005). No significant differences were observed in term of other variables between three tertiles of serum 25-hydroxyvitamin D [25(OH)D] (Table 1).

**Table 1:** Characteristics of the sample per tertile of serum 25(OH)D.

Variables	T1 (≤15.0 ng/ml)	T2 (15.1–25.0 ng/ml)	T3 (≥25.1 ng/ml)	*P*
Gender (M/F)	31/35	32/24	28/6	0.003
Age (years)	13.8 ± 2.7	14.0 ± 2.0	13.9 ± 2.4	0.910
BMI (kg/m^2^)	18.8 ± 2.8	18.9 ± 2.9	18.2 ± 2.6	0.441
Education (years)	9(7, 10)	9(8, 11)	9(6, 10)	0.103
Single-parent fmily (yes)	4(6.1)	2(3.6)	2(5.9)	0.794
Sibling (yes)	29(39.1)	20	10(34.8)	0.336
Dialysis mode (HD/PD)	42/24	32/24	21/13	0.760
Dialysis duration (months)	25(20, 28)	24(20, 26)	22(18, 28)	0.251
Number of hospitalization	4(2, 7)	5(2, 8)	6(2, 7)	0.797
Hemoglobin (g/dl)	10.44 ± 1.15	10.21 ± 1.30	10.62 ± 1.20	0.287
Albumin (g/dl)	3.76 ± 1.21	3.56 ± 1.28	3.78 ± 1.25	0.606
Calcium (ng/ml)	9.67 ± 1.39	9.19 ± 1.40	9.52 ± 1.43	0.169
Phosphate (mg/dl)	5.56 ± 1.28	5.16 ± 1.35	5.41 ± 1.32	0.241
iPTH (pg/ml)	361.15 ± 64.66	280.81 ± 97.19	276.03 ± 125.43	<0.001
SCARED scores	29.9 ± 7.0	25.6 ± 10.2	24.0 ± 11.7	0.005
Antihypertensive medication	57(86.4)	45(80.4)	26(76.5)	0.436

Data are expressed as number (percentage) or means (±SD) or medians (25th, 75th percentiles). Abbreviations: SCARED, Screen for Child Anxiety Related Emotional Disorders; BMI, body mass index; HD, hemodialysis; PD, peritoneal dialysis; 25(OH)D, 25-hydroxyvitamin D; iPTH, intact parathyroid hormone.

### Group Differences in Demographic and Clinical Variables

We found that dialysis patients had higher anxiety scores when compared to control subjects (27.1 ± 9.6 vs. 22.8 ± 10.1, *P* = 0.001). Of the 156 patients who formed the study sample, 110(70.5%, 61 boys, 49 girls) presented with anxiety. Vitamin D deficiency was observed in 34.0% of the patients (n = 43), and vitamin D insufficiency, in 48.7% (n = 76). There were no significant differences in mean age and gender between patients and healthy volunteers. No between-season sampling differences were fond for serum levels of 25(OH)D among dialysis patients. Serum levels of 25(OH)D were significantly lower in patients with anxiety than in normal controls (19.4 ± 10.3 vs. 38.6 ± 15.5 ng/ml, *P* < 0.001). Patients with anxiety showed markedly lower serum 25(OH)D levels as compared to patients without anxiety (17.5 ± 9.7 vs. 23.3 ± 10.4 ng/ml, *P* = 0.001). There was a negative correlation between serum levels of 25(OH)D and SCARED scores (*r* = −0.243, *P* = 0.002). There was a negative correlation between 25(OH)D and iPTH (*r* = −0.355, *P* < 0.001). No correlation was found between serum 25(OH)D and age, gender, calcium, phosphorus, albumin as well as with hemoglobin (all *P* > 0.05). Additionally, patients with anxiety had higher iPTH levels (329.01 ± 82.13 vs. 277.28 ± 128.73, *P* = 0.003), higher frequency of hospitalizations [6(4, 9) vs. 2.5(1, 4), *P* < 0.001] and longer time on dialysis [25(20, 28) vs. 22(16, 24), *P* = 0.003] (Table 2).

**Table 2:** Demographic, clinical and laboratory characteristics of the sample.

Variables	Subjects with anxiety (SCARED ≥25, n = 110)	Subjects without anxiety (SCARED <25, n = 46)	P
Gender (M/F)	61/49	30/16	0.259
Age (years)	13.9 ± 2.4	13.5 ± 2.5	0.298
BMI (kg/m^2^)	18.6 ± 2.7	19.0 ± 3.1	0.349
Education (years)	9(7, 10)	9(6, 10)	0.367
Single-parent fmily (yes)	6(5.5)	2(4.3)	0.564
Sibling (yes)	43(39.1)	16(34.8)	0.613
Dialysis mode (HD/PD)	68/42	27/19	0.716
Dialysis duration (months)	25(20, 28)	22(16, 24)	0.003
Number of hospitalization	6(4, 9)	2.5(1, 4)	<0.001
Hemoglobin (g/dl)	10.44 ± 1.33	10.29 ± 0.86	0.470
Albumin (g/dl)	3.62 ± 1.22	3.85 ± 1.29	0.293
Calcium (ng/ml)	9.36 ± 1.32	9.71 ± 1.60	0.165
Phosphate (mg/dl)	5.43 ± 1.35	5.30 ± 1.26	0.532
iPTH (pg/ml)	329.01 ± 82.13	277.28 ± 128.73	0.003
25(OH)D			0.002
Tertile 1	56(50.9)	10(21.7)	0.001
Tertile 2	36(32.7)	20(43.5)	0.202
Tertile 3	18(16.4)	16(34.8)	0.011
25(OH)D (ng/ml)	17.5 ± 9.7	23.3 ± 10.4	0.001
Antihypertensive medication	92(84.4)	36(78.3)	0.357

Data are expressed as number (percentage) or means (±SD) or medians (25th, 75th percentiles). Abbreviations: SCARED, Screen for Child Anxiety Related Emotional Disorders; BMI, body mass index; HD, hemodialysis; PD, peritoneal dialysis; 25(OH)D, 25-hydroxyvitamin D; iPTH, intact parathyroid hormone.

### Differences in Vitamin D Level Tertiles of Subjects

Significant differences in 25(OH)D level tertiles of pediatric patients were observed between the patients with anxiety and the patients without anxiety (*P* = 0.002). Indeed, the proportion of patients in the lowest tertile (≤15.0 ng/ml) was significantly higher in the anxiety group (*P* = 0.001), whilst the proportion of patients in the highest tertile (≥25.1 ng/ml) was significantly lower in the anxiety group *(P* = 0.011) (Table 2).

### Independent Characteristics of Anxiety in Dialysis Patients

With all patients taken as a whole, anxiety occurrence taken as a dependent variable and tertile 2 taken as the reference used for 25(OH)D levels in the logistic analysis, 25(OH)D levels (≤15.0 ng/ml) were independently associated with the existence of anxiety in pediatric patients with dialysis (OR 4.186, 95% CI 1.400–12.517, *P* = 0.010). Moreover, the number of hospitalizations was significantly associated with the presence of anxiety in children and adolescents treated with dialysis (OR 1.519, 95% CI 1.280–1.802, *P* < 0.001) (Table 3).

**Table 3:** Characteristics associated with anxiety in dialysis patients.

Variables	OR (95% CI)	*P*
**25(OH)D**		
Tertile 1	4.186(1.400–12.571)	0.010
Tertile 3	0.521(0.172–1.577)	0.249
Gender	1.352(0.538–3.399)	0.521
Age	1.067(0.889–1.281)	0.485
Dialysis duration	0.959(0.889–1.034)	0.276
Number of hospitalization	1.519(1.280–1.802)	<0.001
iPTH	1.003(0.998–1.008)	0.202

25(OH)D, 25-hydroxyvitamin D; HD, hemodialysis; OR, odds ratio; CI, confidence interval; iPTH, intact parathyroid hormone.

## Discussion

In the present study, we found that 70.5% of Chinese patients with dialysis presented with anxiety, which is similar to the findings of previous studies^3^. Compared with adult patients with dialysis, however, the data regarding the prevalence of anxiety among pediatric patients with dialysis remain scarce, thereby limiting the reliability of the reported findings. Our results also demonstrated a correlation between anxiety and the number of hospitalizations in children and adolescents with dialysis, which agrees with the results of earlier studies^3^.

To the best of our knowledge, this is the first study to explore the possible association between serum vitamin D levels and anxiety in children and adolescents on dialysis. We found that low serum levels of vitamin D were significantly associated with and anxiety in children and adolescents with dialysis. As mentioned earlier, a growing body of research has reported lower vitamin D levels in non-dialysis patients with anxiety^8–12^. Animal experiments have demonstrated a profound anxiolytic-like effect of vitamin D treatment in in female rats with long-term estrogen deficiency^14^. Moreover, study of patients with premenstrual syndrome-related mood disorders has shown positive effects of vitamin D supplementation on reduction in anxiety score^17^.

The exact role of vitamin D in the pathophysiology of anxiety remains unknown. As a neurosteroid hormone across the blood–brain barrier^18^, vitamin D exerts its function via binding to VDR and vitamin D activating enzyme 1ahydroxylase, which are broadly present in neuronal and glial cells of the human brain^6,7^. Studies in animals have demonstrated that the VDR-deficient mice shown increased anxiety symptoms^13^, which suggested that the defects in the vitamin D-VDR system may directly result in the development of anxiety. Moreover, vitamin D plays a key role in modulating the secretion of inflammatory cytokines such as tumor necrosis factor-α (TNF-α) and C-reaction protein (CRP)^19^. A growing body of evidence suggests the involvement of cytokines in the process of anxiety by modulating the metabolism of neurotransmitters such as dopamine and serotonin^20–23^. Up-regulation of inflammatory cytokines and neutrophil activation has been found in non-infected pediatric patients with dialysis^24,25^. Therefore, these results suggest that vitamin D might play an important role in anxiety among children and adolescents receiving dialysis.

It is also possible that pediatric patients with anxiety take less vitamin D in the diet and are less exposed to sunlight; therefore, it is the anxiety which “causes” the low levels of vitamin D in pediatric patients receiving dialysis. The most important question in the present study is the nature and direction of the causal association between serum vitamin D levels and anxiety in children and adolescents with dialysis.

Several limitations of the present study should be noted. First, the seasonal variation of vitamin D status makes it preferable to perform all measurements on the same day. Second, the cross-sectional nature of the study did not allow us to explain the causal relationship between vitamin D and in anxiety among children and adolescents with dialysis. Third, subjects in our sample came from one only one clinic, which limited the generalization of the findings of the present study. Finally, the small sample size reduced the statistical power of the study.

## Conclusion

In spite of the limitations mentioned above, our research demonstrates an important association between serum vitamin D levels and anxiety in anxiety among children and adolescents with dialysis. Further prospective studies on larger populations should be encouraged to explore the exact relationship between them, which may provide a novel therapeutic target for anxiety in children and adolescents with dialysis.

## Materials and Methods

### Study design and population

Pediatric patients on chronic hemodialysis (HD) or peritoneal dialysis (PD) at the Dialysis Center of the First Affiliated Hospital of Jiaxing University between 15 May 2013 and 21 June 2016 were included in this cross-sectional study. Eligibility criteria were as follows: (1) Chinese ethnicity; (2) age between 8 and 18 years; (3) receipt of dialysis therapy for at least 3 months; (4) hospitalization within 14 days (exclusive of a hospital stay for HD or PD); (5) the willingness to give informed consent. Exclusion criteria included: (1) subjects with severe visual or auditory impairment or mental retardation; (2) renal transplant recipients; (3) subjects with a history of psychiatric disorders including anxiety and depression; (4) subjects with malignancy/leukaemia, chronic hepatic disease, autoimmune diseases or active infections; (5) subjects having a significant life event unrelated to their renal disease in the past 30 days, such as severe illness of a family member, family structure changes, losing a family member, changing the living place; (6) subjects with osteoporosis or receiving supplementation with ergocalciferol or cholecalciferol. All dialysis patients were maintained on a single dialysis modality. No patient switched. Meanwhile, 100 healthy volunteers without renal impairment, a history of psychiatric disorders, receiving supplementation with ergocalciferol or cholecalciferol, or osteoporosis, were recruited from a health survey. Written informed consents were obtained from all subjects and their parents before enrollment in the present study. The study was approved by the Ethics Committee of the First Affiliated Hospital of Jiaxing University and was conducted in compliance with the Declaration of Helsinki.

### Clinical Variables

Demographic and clinical data including age, gender, body mass index (BMI), education, family structure, dialysis mode, dialysis duration, chemistry parameters and medications, were obtained from participant report and electronic medical records. BMI was calculated as weight (kg)/squared height (m^2^). Blood samples were obtained for all subjects according to a standard protocol between 8 a.m. and 10 a.m. each morning and stored at *−*80 °C until measurement. The blood samples of the majority patients were collected at summer (44.2%) and fall (35.3%). All blood samples of control subjects were collected at the end of August 2015. Routine chemistry variables such as serum calcium, phosphorus, albumin, hemoglobin, and iPTH, were test by standard automated methods. Serum 25(OH)D was chosen to test vitamin D status for all participants because of its widespread clinical application, standardized ranges and testing protocol. Serum 25(OH)D levels were measured by using a competitive protein-binding assay and the intra-assay coefficient of variation was 7–10%. In the present study, serum 25(OH)D levels <10 ng/ml were considered deficiency and levels of 10–30 ng/ml were considered insufficiency. Serum 25(OH)D levels of all patients were divided into three tertiles (≤15.0, 15.1–25.0, and ≥25.1 ng/ml) owing to its skewed distribution. Because of ties, the number of patients in each tertile was not even.

### Assessment of Anxiety

The Chinese version of SCARED^26,27^ was used to assess the presence of anxiety in our study sample. SCARED is a 41-item self-report questionnaire of a broad range of anxiety symptoms that consists of five subscales which parallel the five subtypes of anxiety in the DSM-IV: panic disorder, generalized anxiety disorder, separation anxiety, school phobia, and social phobia. Each item is scored 0 to 2 (0 almost never, 1 sometimes, 2 often) with higher scores reflecting greater anxiety; a total score over 25 indicates the presence of clinically relevant anxiety. The Chinese version of SCARED has been shown to have good reliability and validity^27^.

### Statistical Analysis

Data are presented as mean ± standard deviation (SD) for normally distributed variables, medians (25th, 75th percentiles) for non-normally distributed variables, and number (percentage) for categorical variables. All patients with dialysis were stratified into two groups on the basis of the presence of anxiety (SCARED scores ≥25). Comparisons between the groups were performed using Student *t* test, one-way ANOVA, Pearson’s *χ*^2^ test, Fisher’s exact test or Mann-Whitney *U* on the basis of the variable type and distribution. Associations were estimated using Spearman or Pearson linear coefficients. Binary logistic regression including age, gender, and the variables with *P* < 0.05 in the univariate analysis, was estimated to determine the correlates of anxiety in children and adolescents with dialysis. The abnormally distributed parameters were log-transformed for satisfying the log-linearity assumption. The results were described as adjusted odds ratios (OR) with the corresponding 95% confidence intervals (CI). All analyses were performed by using SPSS 17.0 (Chicago, IL). Significance level was defined as *P* < 0.05.

### Data availability

The datasets analyzed during the current study are available from the corresponding author on reasonable request.

## References

[1] Bakr A (2007). Psychiatric disorders in children with chronic renal failure. Pediatric nephrology.

[2] Fukunishi I, Honda M, Kamiyama Y, Ito H (1993). Anxiety disorders and pediatric continuous ambulatory peritoneal dialysis. Child psychiatry and human development.

[3] Kilis-Pstrusinska K (2013). Anxiety in children and adolescents with chronic kidney disease–multicenter national study results. Kidney & blood pressure research.

[4] Goldstein SL, Gerson AC, Furth S (2007). Health-related quality of life for children with chronic kidney disease. Advances in chronic kidney disease.

[5] Holick MF (2007). Vitamin D deficiency. The New England journal of medicine.

[6] Eyles DW, Smith S, Kinobe R, Hewison M, McGrath JJ (2005). Distribution of the vitamin D receptor and 1 alpha-hydroxylase in human brain. Journal of chemical neuroanatomy.

[7] Eyles DW, Burne TH, McGrath JJ (2013). Vitamin D, effects on brain development, adult brain function and the links between low levels of vitamin D and neuropsychiatric disease. Frontiers in neuroendocrinology.

[8] Wu C (2016). Association Between Serum Levels of Vitamin D and the Risk of Post-Stroke Anxiety. Medicine.

[9] Huang JY (2014). Association of serum vitamin D with symptoms of depression and anxiety in early pregnancy. Journal of women’s health.

[10] Armstrong DJ (2007). Vitamin D deficiency is associated with anxiety and depression in fibromyalgia. Clinical rheumatology.

[11] Kelley L, Sanders AF, Beaton EA (2016). Vitamin D deficiency, behavioral atypicality, anxiety and depression in children with chromosome 22q11.2 deletion syndrome. Journal of developmental origins of health and disease.

[12] Bicikova M (2015). Vitamin D in anxiety and affective disorders. Physiological research.

[13] Kalueff AV, Lou YR, Laaksi I, Tuohimaa P (2004). Increased anxiety in mice lacking vitamin D receptor gene. Neuroreport.

[14] Fedotova, J., Pivina, S. & Sushko, A. Effects of Chronic Vitamin D(3) Hormone Administration on Anxiety-Like Behavior in Adult Female Rats after Long-Term Ovariectomy. *Nutrients* **9**10.3390/nu9010028 (2017). 10.3390/nu9010028 PMC5295072 28054941

[15] Cho HY, Hyun HS, Kang HG, Ha IS, Cheong HI (2013). Prevalence of 25(OH) vitamin D insufficiency and deficiency in pediatric patients on chronic dialysis. Peritoneal dialysis international: journal of the International Society for Peritoneal Dialysis.

[16] Dibas BI, Warady BA (2012). Vitamin D status of children receiving chronic dialysis. Pediatric nephrology.

[17] Tartagni M (2016). Vitamin D Supplementation for Premenstrual Syndrome-Related Mood Disorders in Adolescents with Severe Hypovitaminosis D. Journal of pediatric and adolescent gynecology.

[18] Kesby JP, Eyles DW, Burne TH, McGrath JJ (2011). The effects of vitamin D on brain development and adult brain function. Molecular and cellular endocrinology.

[19] Bellia A (2013). Serum 25-hydroxyvitamin D levels are inversely associated with systemic inflammation in severe obese subjects. Internal and emergency medicine.

[20] Cai W (2005). Interferon-alpha-induced modulation of glucocorticoid and serotonin receptors as a mechanism of depression. Journal of hepatology.

[21] Moron JA (2003). Mitogen-activated protein kinase regulates dopamine transporter surface expression and dopamine transport capacity. The Journal of neuroscience: the official journal of the Society for Neuroscience.

[22] Stewart A (2010). Neurosteroid vitamin D system as a nontraditional drug target in neuropsychopharmacology. Behavioural pharmacology.

[23] Wrzosek M (2013). Vitamin D and the central nervous system. Pharmacological reports: PR.

[24] Polanska B (2014). Elastase, alpha1-proteinase inhibitor, and interleukin-8 in children and young adults with end-stage kidney disease undergoing continuous ambulatory peritoneal dialysis. Archivum immunologiae et therapiae experimentalis.

[25] Cetin N, Sav NM, Karabel D, Yildirim A, Yildiz B (2016). Serum albumin and von Willebrand factor: possible markers for early detection of vascular damage in children undergoing peritoneal dialysis. Clinical and investigative medicine. Medecine clinique et experimentale.

[26] Birmaher B (1999). Psychometric properties of the Screen for Child Anxiety Related Emotional Disorders (SCARED): a replication study. Journal of the American Academy of Child and Adolescent Psychiatry.

[27] Su L, Wang K, Fan F, Su Y, Gao X (2008). Reliability and validity of the screen for child anxiety related emotional disorders (SCARED) in Chinese children. Journal of anxiety disorders.

